# TGF-Beta Induced Erk Phosphorylation of Smad Linker Region Regulates Smad Signaling

**DOI:** 10.1371/journal.pone.0042513

**Published:** 2012-08-06

**Authors:** Chris Hough, Maria Radu, Jules J. E. Doré

**Affiliations:** 1 BioMedical Sciences, Memorial University, St. John's, Newfoundland, Canada; 2 Cancer Biology Program, Fox Chase Cancer Center, Philadelphia, Pennsylvania, United States of America; University of Edinburgh, United Kingdom

## Abstract

The Transforming Growth Factor-Beta (TGF-β) family is involved in regulating a variety of cellular processes such as apoptosis, differentiation, and proliferation. TGF-β binding to a Serine/Threonine kinase receptor complex causes the recruitment and subsequent activation of transcription factors known as smad2 and smad3. These proteins subsequently translocate into the nucleus to negatively or positively regulate gene expression. In this study, we define a second signaling pathway leading to TGF-β receptor activation of Extracellular Signal Regulated Kinase (Erk) in a cell-type dependent manner. TGF-β induced Erk activation was found in phenotypically normal mesenchymal cells, but not normal epithelial cells. By activating phosphotidylinositol 3-kinase (PI3K), TGF-β stimulates p21-activated kinase2 (Pak2) to phosphorylate c-Raf, ultimately resulting in Erk activation. Activation of Erk was necessary for TGF-β induced fibroblast replication. In addition, Erk phosphorylated the linker region of nuclear localized smads, resulting in increased half-life of C-terminal phospho-smad 2 and 3 and increased duration of smad target gene transcription. Together, these data show that in mesenchymal cell types the TGF-β/PI3K/Pak2/Raf/MEK/Erk pathway regulates smad signaling, is critical for TGF-β-induced growth and is part of an integrated signaling web containing multiple interacting pathways rather than discrete smad/non-smad pathways.

## Introduction

Transforming Growth Factor β (TGF-β) is the prototypic member of a family of structurally related cytokines that control a myriad of cellular functions. TGF-β elicits its cellular responses by signaling through a receptor complex of serine/threonine kinase type I (TβRI) and type II (TβRII) receptors [1], [2]. Ligand binding induced signal transduction through this receptor complex results in receptor mediated (R-) smad2 and/or smad3 phosphorylation. This phosphorylation at the C-terminal SSXS motif of smad2/3 allows them to complex with the common mediator (Co-) smad4 [3], [4], translocate into the nucleus, and regulate target gene expression [5], [6]. Although both mesenchymal and epithelial cells contain the canonical TGF-β/smad signaling cascade, epithelial cell types are growth inhibited, whereas mesenchymal cells are growth stimulated by TGF-β suggesting a fundamental mechanistic difference in TGF-β signaling between cell types, supplimental to the smad signaling cascade. This has lead to the nomenclature of smad and non-smad or smad-dependant and independent signaling cascades.

There have been a number of these “non-smad” signaling pathways described including Erk, Jnk, ROCK, and more recently, p21-activated kinase-2 (Pak2; [7]–[11]). In phenotypically normal cell lines (neither virally transformed nor cancer derived), TGF-β regulation of Pak2 activity was found to be stimulated through cdc42/Rac1 and inhibited by Merlin/Erbin [10], [11]. Pak2 is specifically activated by TGF-β only in mesenchymal cells, as the result of phosphatidylinositol 3-kinase (PI3K) activation and may be associated with TGF-β activation of Ras [10], [12], [13]. Conversely, normal epithelial cells appear to inhibit Pak2 activation through an inability to activate PI3K and/or by directly inhibiting Pak2 through Merlin/Erbin [11]. Functionally, PAKs regulate apoptosis, cell motility and cytoskeletal rearrangement [14]. Relevant to this study, Paks have been implicated in mitogen activated protein kinase/extracellular signal regulated kinase (MAPK/Erk) signaling cascades as potential MAP kinase kinase kinase kinases [15] by regulating the activity of both c-Raf and MEK1 [16], [17]. Classically, with tyrosine kinase receptors, activation of Ras [18], [19] results in activated Raf, which activates MEK1/2, followed by Erk activation. However, Ras independent mechanisms of Erk activation have been described for both erythropoietin (Epo; [20]) and platelet derived growth factor (PDGF; [21]), suggesting different pathways lead to Erk activation.

Although cross-talk between Erk and smad signaling was described over a decade ago [7], [18], [22], the relationship and mechanism by which this occurs is still unknown. Within the linker region domains of smad2 and smad3 are several potential Erk phosphorylation sites [23], [24]. However, these same sites have also been implicated in smad regulation by the cyclin dependent kinases, CDK8 and 9 [25]. The phosphorylated linker region, has also been shown to both inhibit smad nuclear translocation and signaling [18], [24], [26]–[28] and enhance smad mediated transcriptional activity [7], [23], [25], two mutually exclusive functions.

To address this controversy, in this study we further refine the mechanism for cell type specific TGF-β activation of Erk. We show that via PI3K, Pak2 activation results in Erk activation in untransformed cells with endogenous levels of signal transduction proteins. We also show that this activated Erk phosphorylates smads within their linker regions, resulting in the maintenance of smad mediated transcriptional activation, thus demonstrating integration of the Erk and smad pathways, both under the direct control of TGF-β.

## Materials and Methods

### Cell Culture

All cell lines used were maintained in high glucose Dulbecco's Modified Eagle Medium (DMEM; Invitrogen, Carlsbad, CA) and purchased from American Type Culture Collection repository (Mannassas, VA; NIH-3T3, CRL-1658; Mv1Lu, CCL-64; HEK-293A, CRL-1573; NMuMG, CRL-1636). The murine embryonic fibroblast cell line, AKR-2B, was grown in DMEM supplemented with 5% Fetal Bovine Serum (FBS; PAA Labs Inc, Etobicoke, ON)), while NIH-3T3 cells were grown in DMEM supplemented with 10% Newborn Calf Serum (NBCS; Invitrogen, Carlsbad, CA). Pak2 flox/flox MEF parental cell line and the Cre/Pak2 knockout derivative (kind gift of Dr. Jonathan Chernoff, Fox Chase Cancer Centre, OH) were maintained in DMEM supplemented with 10% FCS, as were Mv1Lu epithelial cells, while NMuMG growth media also contained 10 µg/ml bovine Insulin (Sigma Biochemicial, St. Louis, MO) and 5 ng/ml EGF (Cell Signaling Technologies; Pickerington, ON). All buffer salts, bovine serum albumin (BSA) and acrylamide were purchased from ThermoFisher Biotechnology.

### Protein Analysis

Mesenchymal cell lines were plated 24 h prior to serum depletion (0.1% NBCS/DMEM) 18 h prior to experimentation. Epithelial cell lines were treated approximately 18 hours after plating (Mv1Lu), or medium was replaced with 10% FCS/DMEM, 18 hours prior to treatment to remove interference from supplemental growth factors (NMuMG). Cells were stimulated with 2 ng/ml TGF-β1 (US Biological, Swampscott, MA) for indicated time periods. Inhibitors to PI3K (LY294002; Upstate Biologicals; Billerica, MA), MEK1/2 (U0126; Cell Signal Technologies; Pickerington, ON), proteosomal inhibitor (MG132; Tocris Bioscience; Ellisville, MO) were used at 10 µM dissolved in DMSO, the TGF-β receptor inhibitor (LY364947, Tocris Bioscience; Ellisville, MO) was used at 500 nM and also dissolved in DMSO, while the Ras inhibitor (FPT II; EMD Biosciences, Gibbstown, NJ) was used at 20 µM dissolved in water.

Total cellular protein was obtained by lysing cells with RIPA lysis buffer [29] and quantified for total protein by BCA assay with a standard curve generated using a BSA standard (Pierce/ThermoFisher Scientific; Rockford, IL). Aliquots of equivalent total protein were separated by polyacrylamide gel electrophoresis and transferred to PVDF (Millipore; Billerica, MA) or Nitrocellulose (Pall Life Sciences; Pensacola, FL) membranes prior to antibody detection of each specific protein of interest. Primary antibody binding was detected using a goat anti-rabbit IgG-Horseradish peroxidase secondary antibody (Santa Cruz Biotechnology: Santa Cruz, CA), visualized with Supersignal West Pico Chemiluminscent Substrate (Pierce/ThermoScientific; Rockford, IL) by exposing Hyperfilm (GE Healthcare Bio-Sciences; Piscataway, NJ). All primary antibodies used were from Cell Signal Technologies and included p44/22 MAP Kinase, Phospho-p44/22 (Thr202/Tyr204), Phospho-Akt (Ser473), and Akt, Phospho-smad2 (Ser245/250/255), Phospho-smad2 (Ser465/467), smad2, Phospho-c-Raf (Ser338), and Pak2. Anti-phospho-smad3 (Ser423/425) antiserum was the kind gift of Dr. Ed Leof (Mayo Clinic, Rochester, Minnesota; [10]).

### Cell Assays

The thymidine incorporation assay was a modification of previously described methods [30]. Briefly, NIH 3T3 cells were plated at 40 000 cells/well in a 24 well plate and allowed to attach for 24 h. Cells were then serum depleted for 24 h prior to treatment (TGF-β, 5 ng/ml), supplemented with or without U0126 for 18 hours. Tritiated Thymidine (1 µCi; PerkinElmer; Shelton, CT) was added to each well and incubated for 2 h at 37°C. Macromolecular incorporated radioactivity was precipitated with ice cold 10% trichloroacetic acid, solubilized (0.2 N NaOH, 200 µg/ml ssDNA) then quantified using a Beckman Coulter LS6500 Liquid Scintillation Counter.

Dominant negative Pak2-EGFP (Ad-dnEGFP-Pak2) fusion protein and control EGFP (Ad-EGFP) expressing adenoviruses were generously provided by Dr. Ed Leof (Mayo Clinic, Rochester, Minnesota) and Dr. Mark Natchigal (Dalhousie University, Halifax, NS), respectively. Adenovirus constructs were amplified by infecting HEK-293A cells. Fibroblast cells (AKR-2B or NIH 3T3) were plated 8 h prior to infection with the indicated virus at a multiplicity of infection (MOI) of 125∶1 for 24 h, then either serum depleted for pathway analysis (AKR-2B), or replated for thymidine incorporation assays (NIH 3T3).

Smad signaling duration was determined using a variation of a pulse-chase treatment of AKR-2B fibroblasts. Cells were plated and serum depleted as previously described. Cells were pretreated with U0126 (10 µM) 30 minutes prior to the addition of TGF-β (2 ng/ml) for 10 minutes. Medium was replaced with prewarmed medium (37°C, 0.1%NBCS/DMEM) with or without supplimentation of U0126. The indicated treatment times are relative to the addition of TGF-β to the medium. Protein lysates were prepared as previously described while RNA was isolated from separate experiments using Trizol (Invitrogen; Carlsbad, CA) following the manufacturer's protocol. Total RNA was evaluated and quantified using a 2100 Bioanalyzer (Agilent Technologies; Waldbronn, Germany). Samples with a RIN value greater than 8.5 were used. Target gene mRNA levels were evaluated using TaqMan assays on-demand (Applied Biosystems; Foster City, CA) with 18S rRNA as the internal standard, Mm00435860_m1 for plasminogen activator-1 (PAI-1), or Mm00484742_m1 for Smad 7. Relative expression levels were determined using the ΔΔCt method [31] where target gene expression was evaluated relative to 18S rRNA levels. Untreated control levels were arbitrarily set at 1 to which the treatment groups were compared. Differences between mean levels of treatment group's target gene expression were analyzed with Graphpad Prism software by 1-way ANOVA using a Tukey's Multiple Range test for Post-hoc analysis. Differences in band intensities of western blots were determined using Image J software to define the amount of phospho-proteins, relative to their respective loading control. Decay rates for C-terminal phospho-smad2 and 3 were determined using Graphpad Prism software to calculate exponential decay curves for each experiment (smad 2, n = 3; smad 3, n = 4). Exposure differences between experiments were equalized by setting the 60 min. value at 100% and calculating the intensities relative to this value.

### Immunocytochemistry

NIH 3T3 cells were plated on 4 chamber slides (Lab-Tek; ThermoFisher Scientific; Rockford, IL), treated as previously described. Cells were fixed with 4% Paraformaldehyde/PBS, permeabilized with 0.2% Triton X-100, blocked [PBS, 5% BSA, 10% normal goat serum (Sigma/Aldrich Chemical Company; St. Louis, MO)], prior to incubation in primary antibody overnight at 4°C in a humidified chamber. Immune complexes were detected with Rhodamine X conjugated goat anti-rabbit secondary antibody (BioCan Scientific; Mississauga, ON), and coverslipped with Vectashield (Vector Laboratories; Burlingame, CA). Photomicrographs were obtained using a Leica DMII microscope with a TX2 filter cube and Open Lab software. Digital images were all adjusted for size, contrast and brightness equally using Photoshop software to preserve the original relative magnification and intensities.

## Results

### TGF-β Activates Erk in a Cell Type Specific Manner

**Figure 1:**
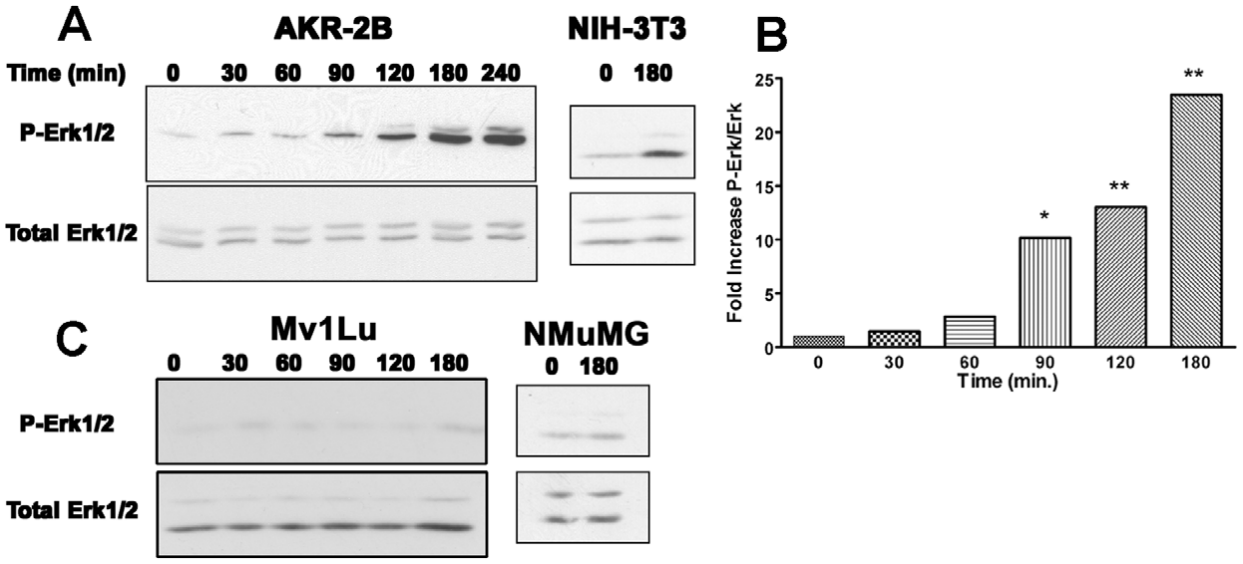
Cell type specific activation of Erk. (**A**) AKR-2B and NIH 3T3 fibroblast were treated with TGF-β (2 ng/ml) for times ranging from 0 to 4 h. Cell lysates were probed with antibodies specific to phospho-Erk (P-Erk). Blots were then stripped and reprobed for total Erk as a loading control. Typical results are shown representing four independent time course experiments. (**B**) Graph of densitometric analysis of western blots from time courses from 0–3 h (n = 4) of phospho-Erk. Shown is the mean fold increase of Phospho-Erk relative to total Erk for each time point with the 0 time set as 1. Statistically significant change from 0 time is noted as, (*) P<0.05 and (**) P<0.01. (**C**) Mv1Lu and NMuMG epithelial cell lines were treated with TGF-β (2 ng/ml) for times ranging from 0 to 3 h. Cell lysates were probed with antibodies specific to phospho-Erk (P-Erk). Blots were then stripped and reprobed for total Erk as a loading control. Triplicate blots were performed on three independent time course experiments with typical results shown.

Since TGF-β has been known to stimulate fibroblast replication and had been shown to activate PI3K [10], [12] we wished to further define activation of the Erk pathway in normal (untransformed) cell lines. Temporal changes in Erk phosphorylation as the result of fibroblast cell lines treated with TGF-β over the course of 4 h were determined (Figure 1). Taking into account protein loading (total Erk) between lanes, we found Erk phosphorylation increased significantly above background between 60–90 minutes after TGF-β addition (Figure 1B). Previous reports have shown Erk activation at earlier time points [7], [13]. We likewise saw an increase in phospho-Erk earlier, but only when cells were allowed to cool when taken from the incubator during addition of TGF-β. Cells maintained near 37°C, demonstrated no significant activation of Erk prior to the 60–90 minute window. Earlier activation was consistent with Erk signaling being indicative of an induced stress response [32], [33]. Since TGF-β activation of PI3K has been shown to be cell type dependent [10], [12] the previous experiment was repeated using Mv1Lu and NMuMG epithelial cells. No increase in Erk phosphorylation was identified at any time point (Figure 1C) following TGF-β treatment. Together these results confirm that activation of Erk upon TGF-β treatment occurs in phenotypically normal cells of mesenchymal origin, but not epithelial cells.

### TGF-β Mechanism of Erk Activation

**Figure 2:**
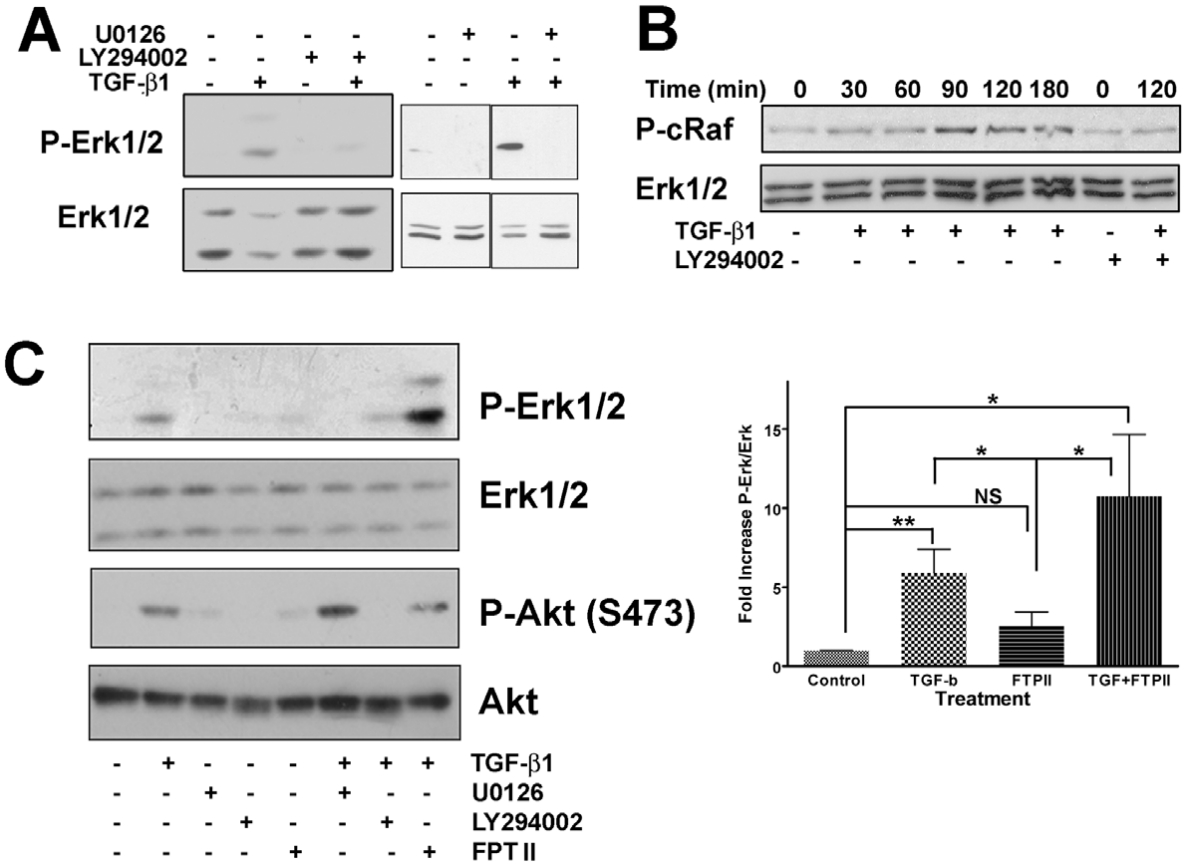
Erk is activated in fibroblasts via the PI3K/c-Raf/MEK pathway. (**A**) AKR-2B fibroblasts were treated with the PI3K inhibitor LY294002 or MEK inhibitor U0126 (10 µM) 30 minutes prior to addition of TGF-β (2 ng/ml) for 2 h. Cell lysates were probed with an antibody specific to phospho-Erk, blots were then stripped and reprobed for total Erk as a loading control. (**B**) AKR-2B fibroblasts were treated with TGF-β (2 ng/ml) for the indicated times. Cells were also treated with LY294002 (10 µM) 30 minutes before TGF-β was added for 2 h. Blots were probed using an antibody specific to phospho-c-Raf (Ser338), and total Erk as a loading control. The loading control blot was obtained using the lower molecular mass portion of the same blot. (**C**) AKR-2B fibroblasts were treated with LY294002, U0126 or Ras inhibitor FPTII (10 µM or 20 µM, respecitively) 30 minutes prior to addition of TGF-β (2 ng/ml) for 2 h. Cell lysates were probed with antibodies specific to phospho-Erk or phospho-Akt (S473) with the blots stripped and reprobed for the corresponding total protein as a loading control. Comparisons of the relative intensity of bands of Phopho-Erk relative to total Erk loading control was expressed as fold increase relative to untreated control (set at 1). Statistical analysis was used to determine if treatments were not significant (NS), or significant at P<0.05 (*) or P<0.01 (**). Analysis was performed on four independent blots and the mean values (±SEM) shown.

Having shown Erk activation, via TGF-β, as a cell type specific response, we next wanted to confirm the mechanism through which the TGF-β signal was propagated. Previous studies have shown that TGF-β induced activation of Pak2 and/or Ras [10], [12], [13], we sought to identify the pathway resulting in Erk activation. Using LY294002, a specific inhibitor of PI3K, Erk phosphorylation was inhibited (Figure 2). Additionally, the MEK1/2 inhibitor U0126, blocked Erk phosphorylation, demonstrating that not only is PI3K necessary but also the MAPKK, MEK is involved in TGF-β activation of Erk. There was also a temporal increase in c-Raf phosphorylation (Figure 2B) dependent on TGF-β stimulation and subsequent to PI3K activation as shown by the phosphorylation of S338, a known Pak activation site [34], [35] and its inhibition by the PI3K inhibitor LY294002. In the canonical Erk pathway, activated Ras initiates a cascade resulting in Erk activation. Using small molecular weight inhibitors, we evaluated the activation of two different PI3K activated pathways, Erk and Akt (Figure 2C). Although both pathways were activated by TGF-β through PI3K, only Erk phosphorylation was sensitive to U0126 and neither pathway was inhibited by the Ras farnysylation inhibitor, FPT II [36]. TGF-β induced Erk phosphorylation was significantly increased, above control levels (untreated and FTPII), with no significant difference found between the two TGF-β treated groups (TGF-β alone and TGF- β+FTPII).

**Figure 3:**
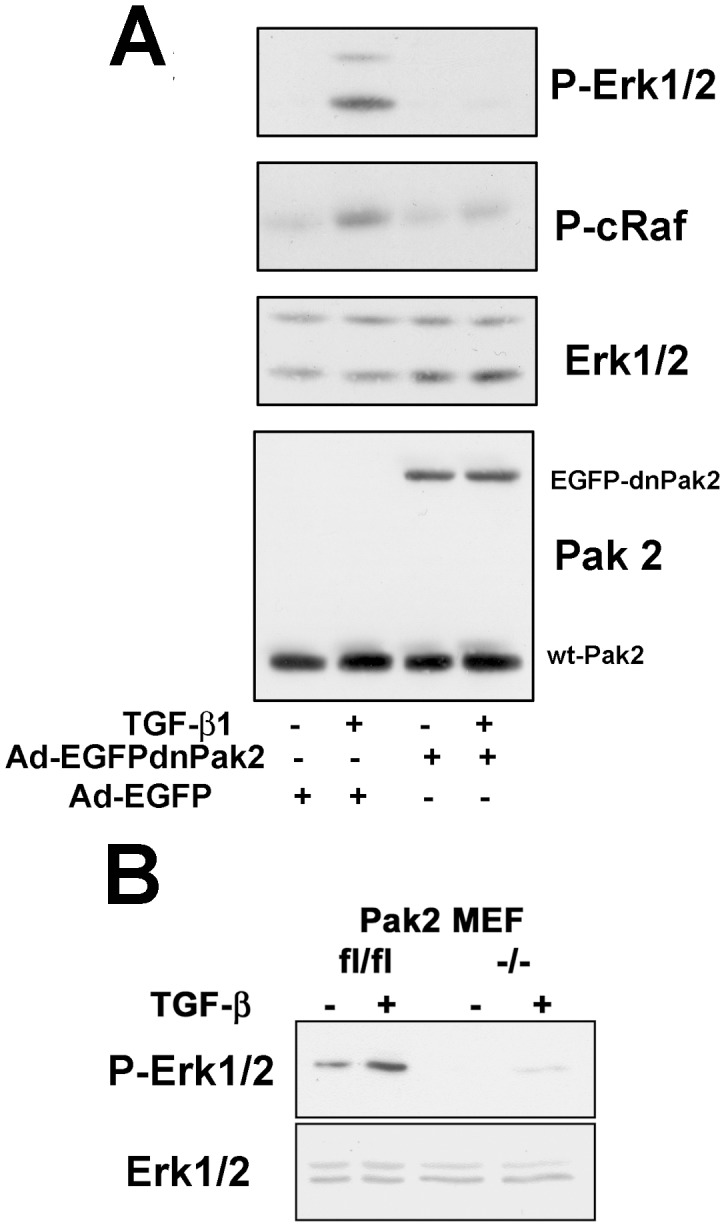
Erk activation requires activation of Pak2 and c-Raf. (A) AKR-2B fibroblasts were infected at an MOI of 1∶125 with adenovirus containing either dominant-negative PAK2 (Ad-EGFPdnPAK2) or Ad-EGFP as a negative control. Cells were treated with TGF-β (2 ng/ml) for 2 h prior to lysis. Cell lysates were then probed for phospho-Erk, and phospho-c-Raf (Ser338). The phospho-Erk1/2, and the stripped/reprobed total Erk loading control blot was obtained using the lower molecular mass portion of the same blot as that for P-cRaf. The same cell lysates were analyzed on a separate blot for PAK2 to confirm expression. (B) Cell lysates were analyzed for phospho-Erk and total Erk levels, in MEF cells that contained the Pak2 gene with flanking flox sites and a MEF cell line derived from this parental line in which Cre recombinase had been used to excise the Pak2 gene, following treatment with or without TGF-β for 2 h.

Since inhibition of Ras farnysylation did not appear to effect TGF-β induced Erk activation, and previous data had shown Pak to be downstream of PI3K and phosphorylate Raf [34], [35], we wanted to determine where Pak2 acted within this signaling pathway. Initially we assessed S338 phosphorylation of c-Raf in fibroblasts expressing a dn-Pak2. When fibroblasts were treated with TGF-β, both c-Raf and Erk phosphorylation was dramatically reduced (Figure 3A). Additionally, a dramatic reduction in phosphorylation of Erk (Figure 3B) was seen when Pak2 was knocked out in MEFs. The low levels of Erk activation in both dnPak2 expressing fibroblasts and Pak2 KO-MEFs, suggests Erk activation can occur via Ras, albeit at a greatly reduced amount. This data is consistent with the hypothesis that TGF-β induced phosphorylation of Erk primarily follows the pathway of PI3K/Pak2/c-Raf/MEK/Erk, with a secondary contribution of Ras, similarly to that described for PDGF [21].

### Smad2 Linker Region is Phosphorylated by Erk

**Figure 4:**
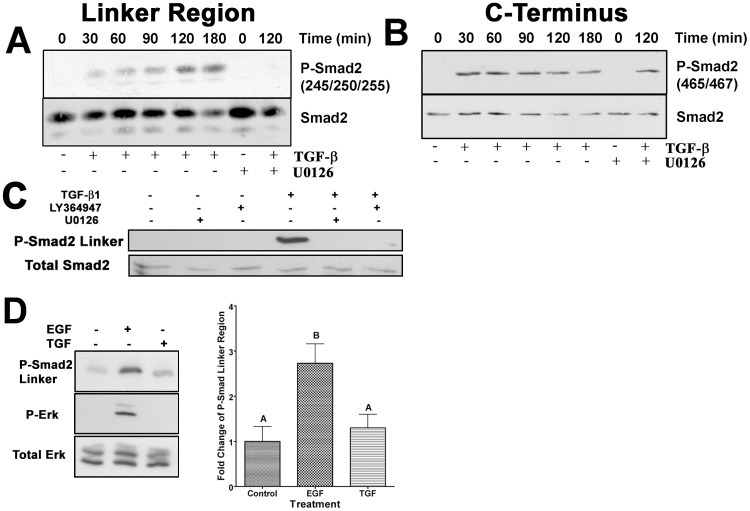
TGF-β directs Erk phosphorylation of Smad2 linker region. (**A**) Western blots from AKR-2B fibroblast cell lysates treated with TGF-β (2 ng/ml) for the indicated time periods with or without U0126 (10 µM) for 120 min. Blots were probed for smad2 phosphorylated within the linker region at S245, 250 and 255, striped and reprobed for total smad2 to demonstrate each loading. (**B**) Receptor mediated phosphorylation of smad2 was also determined in the same samples using antibodies specific to C-terminal Ser 465/467. The blots were stripped and reprobed for total smad2 to demonstrate similar loading of all samples. Representative western-blots are shown with each time course performed in triplicate with consistent results. (**C**) Western blot and relative quantification of smad2 linker region phosphorylation in AKR-2B fibroblasts treated for 30 min. with or without TGF-β (2 ng/ml) or EGF (50 ng/ml). P-Erk blots are also shown to indicate Erk activation. Intensity of P-smad2 linker region band was determined and expressed graphically as fold increase (using total Erk band intensity as the loading control) relative to untreated control values for each experiment. The mean values of three independent experiments are shown (±SEM). Letters above each column indicate the different statistically significant (P>0.05) groupings. (**D**) Representative western blots showing phosphorylation of smad2 linker region of AKR-2B fibroblasts treated for 120 min. with or without TGF-β (2 ng/ml) and/or inhibitors U0126 or LY364947. Blots were stripped and reprobed for total smad 2 as a loading control.

Having shown a direct TGF-β/Erk signaling pathway, our next goal was to show an association existed between Erk and smad signaling, both under the direct control of TGF-β. AKR-2B fibroblasts were treated with TGF-β and probed for phosphorylation of smad2 at the Erk phosphorylation sites (Figure 4A; Ser 245, 250, and 255). We observed a temporal increase in smad2 linker region phosphorylation that was dependant on MEK activitivation of Erk, as treatment with U0216 abolished linker region phosphorylation (Figure 4A,C). Similarly, inhibition of TGF-β receptor kinase activity (LY364947) blocked smad2 linker phosphorylation (Figure 4C). Smad2 phosphorylation showed prominent C-terminal phosphorylation (Ser465/467) at all times tested, independent of MEK activity (Figure 4B; TGF-β+U0126). The linker phosphorylation seen at the 30 min. time point (Figure 4A) was not significantly above background levels (0 min.). However, rapid activation of Erk by EGF did result in significant smad2 linker phosphorylation (Figure 4D). Together this data indicates TGF-β stimulation causes phosphorylation of smad 2 via two distinct mechanisms, within the linker region, through Erk, and at the C-terminus, by TGF-β receptors.

**Figure 5:**
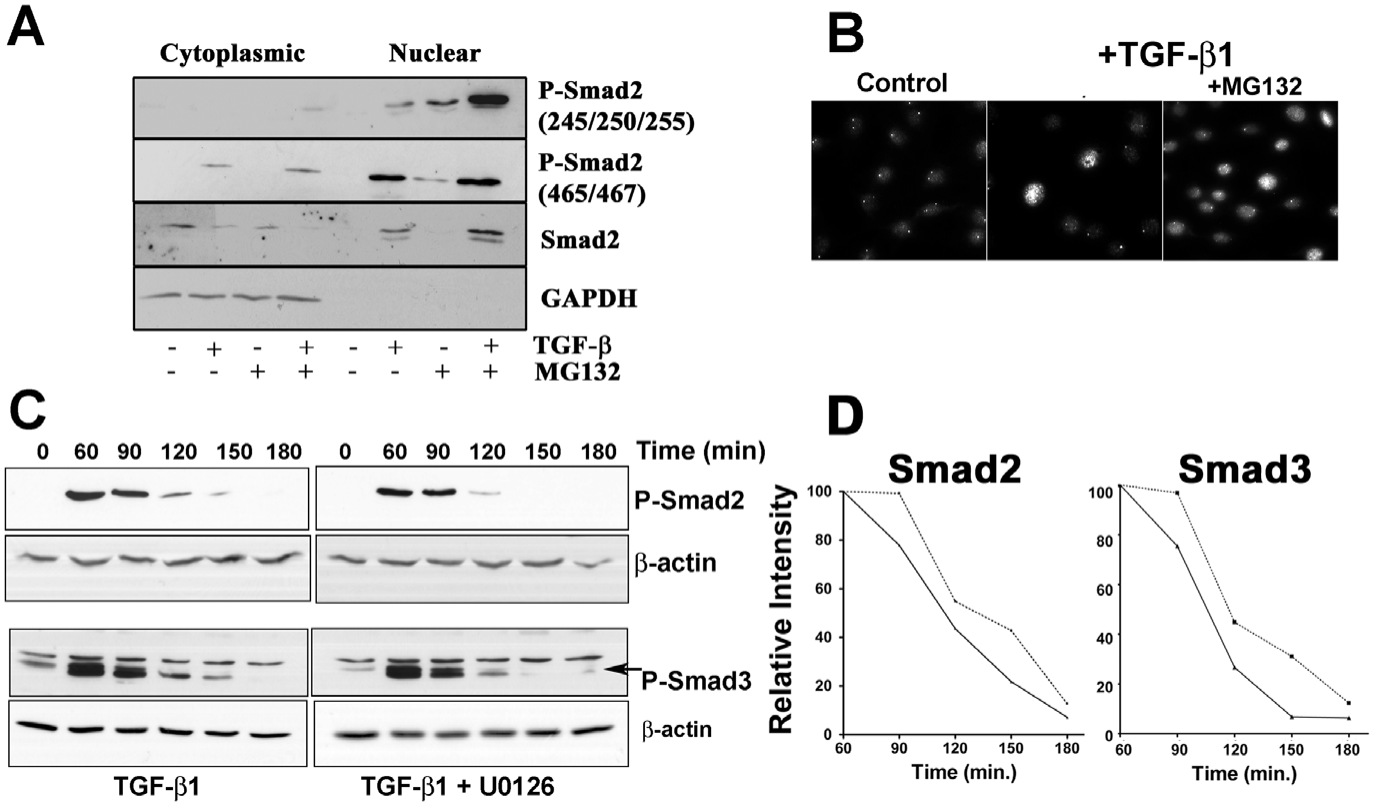
Nuclear Smad levels are controlled by the proteasome and activated Erk. (**A**) AKR-2B fibroblasts were treated for 3 h with TGF-β (2 ng/ml) with or without MG132 (10 µM), 30 minutes prior to TGF-β addition. Nuclear and cytoplasmic fractions were isolated and probed for smad2 linker region phosphorylation (245/250/255), or receptor phosphorylation (465/467). Linker phosphorylation blots were stripped and reprobed for total smad2 as a loading control, while receptor phosphorylated blots were stripped and reprobed for GAPDH to monitor the presence of cytoplasmic protein in the nuclear fraction. (**B**) Photomicrographs of NIH 3T3 fibroblasts treated with TGF-β (2 ng/ml) for 3 h with or without MG132 (10 µM) added 30 minutes prior to TGF-β treatment. Cells were incubated with phospho-smad2 (S245/250/255) linker antibody and specific immune complexes detected using Rhodamine X conjugated secondary antibody. (**C**) Cell lysates from AKR-2B fibroblasts pulsed for 10 minutes with TGF-β (2 ng/ml) with or without U0126 (10 µM) were probed for receptor phosphorylated smad2 and smad3. Blots were stripped and reprobed for β-actin as a loading control. (**D**) The density of phospho-smad2 and 3 bands for each time point relative to its β-actin control were determined. The mean values for each time point (n = 3 for smad 2, n = 4 for smad 3) are displayed with the solid line representing the curve for TGF-β+U0126 and the dotted line representing TGF-β treatment.

Although these results indicate a direct cross-talk between smad2 and Erk, in light of the controversy between the Erk and smad linker region phosphorylation, we wished to examine their spatial relationship. Since smad2 is a transcription factor that translocates quickly into the nucleus following receptor mediated phosphorylation, we fractionated TGF-β treated fibroblasts into cytoplasmic and nuclear fractions and examined smad2 phosphorylation. Linker phosphorylation was seen primarily in the nuclear fraction (Figure 5A). Similarly, receptor phosphorylated smad2 was primarily in the nucleus, with small amounts in the cytoplasmic fraction, following TGF-β treatment. Total smad2 was present in both the cytoplasm and the nucleus. Since our concern was cytoplasmic protein contamination of the nuclear fractions, both cytoplasmic and nuclear fractions were probed for GAPDH to demonstrate purity of nuclear fractions relative to cytoplasmic protein contamination (Figure 5A).

In order to substantiate our findings, immunofluorescent localization of phospho-smad2 linker region was determined. Nuclear localization of phospho-smad2 linker region was observed, in agreement with previous biochemical data that phosphorylated smad2 linker region localizes primarily in the nucleus of fibroblasts (Figure 5B). These data suggests Erk phosphorylation of the smad2 linker region is likely limited to a specific subcellular location via TGF-β induction of both smad C-terminal phosphorylation/translocation and activation of the PI3K/Pak2/Erk pathway.

### Smad linker region function

To ascertain the function of linker region phosphorylation of smads, initially we treated fibroblasts with the proteosomal inhibitor MG132 with and without TGF-β (Figure 5A,B). Nuclear levels of both receptor and linker region phosphorylated smad 2 increased with inhibition of proteosomal degradation, consistent with previous data showing nuclear proteosomal degradation of smads [37], but suggested that linker region phosphorylation may be associated with directing smads to proteosomal degradation [28]. However, TGF-β stimulated fibroblasts treated with U0126 alone or with MG132 did not show an increase in C-terminal phospho-smad2 or 3, even after 3 h, as would be expected if Erk mediated linker phosphorylation targeted smads for proteosomal degradation (data not shown). To the contrary, treatment of fibroblast with TGF-β, in the absence or presence of active Erk (Figure 5C), demonstrated that linker region phosphorylation (TGF-β alone) resulted in sustained levels of smad 2 and 3 (C-terminal phosphorylated), suggesting that linker phosphorylation is associated with an increased stability of C-terminal phospho-smads 2 and 3. Using the band densities of multiple experiments, we calculated decay rates for the C-terminal phospho-smad proteins (Figure 5D). The time to decrease the signal intensity by 50% differed between treatment groups for both smad 2 and 3 (116 vs. 135 min. for smad2, and 100 vs 127 min. for smad3; P<0.05, for TGF-β+U0126 vs. TGF-β alone, respectively). Although only linker region phosphorylation of smad2 is shown in previous figures (Figures 4 and 5), there are equivalent putative Erk phosphorylation sites found on smad 3 [24] but the equivalent phospho-linker region antibody for smad 3 is not commercially available. Based on the data shown in figure 5C, smad 3 linker region is similarly phosphorylated by Erk, in that U0126 has the same effect on inhibiting the decay of C-terminal phosphorylated smads 2 and 3.

**Figure 6:**
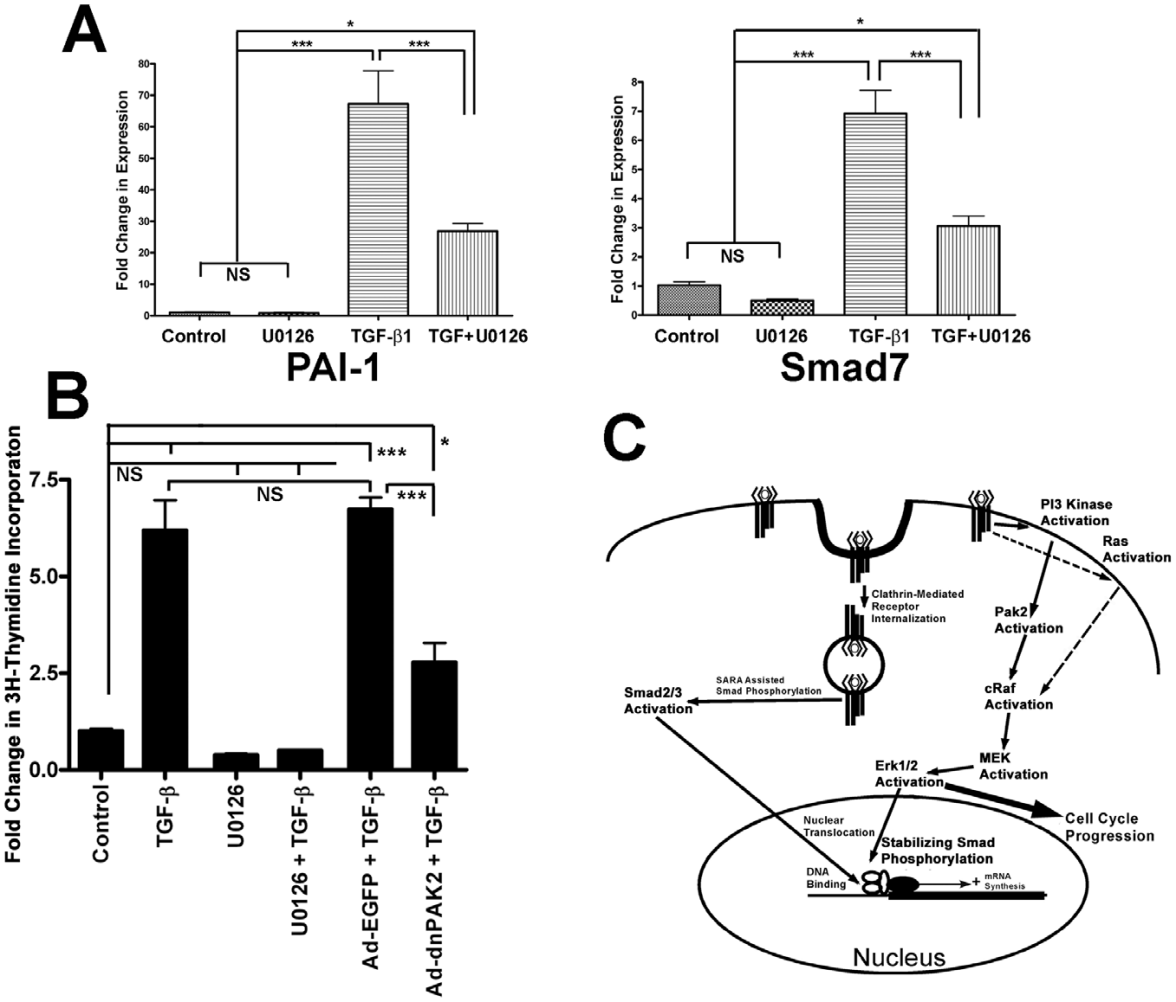
Erk activity is integral for TGF-β signaling and inducing growth in fibroblasts. (**A**) Fold change in expression levels of two smad regulated genes, PAI-1 and smad7, relative to untreated controls levels are shown from AKR-2B fibroblasts treated for 10 minutes with or without TGF-β (2 ng/ml), with or without U0126 (10 µM) for 3 h. The mean values (±SEM) of six independent experiments are shown with statistical evaluation indicating differences between groups as (NS) not significant, (*) P<0.05, (***) P<0.001. (**B**) Thymidine incorporation was determined in serum deprived NIH 3T3 cells treated with TGF-β (5 ng/ml) with or without infection with Ad-dnPAK2 or Ad-EGFP (MOI = 125∶1) or U0126 (10 µM) prior to treatment. The effect of treatment is expressed as a fold change in thymidine incorporation relative to untreated cells (control = 1). The mean (±SEM) of triplicate assays is shown with statistical evaluation indicating differences between groups as previously describe. (**C**) Schematic representation of TGF-β signaling pathways in fibroblasts, indicating the proposed interactions and their subcellular locations. Arrows represent a functional link between the subsequent proteins, not necessarily a direct interaction.

Since nuclear localized, C-terminal phospho-smads bind DNA and regulate gene expression [38], this implies that increased stability would likely increase duration of function. By transiently treating fibroblasts with TGF-β (10 min.), we wished to assess the stability of smad mediated transcription. The 3 h time point chosen to assess mRNA stability was based on the differences in western blot phospho-smad densities in figure 5C. As shown in figure 5C/D, smads remained phosphorylated longer when Erk was active. Likewise, smad mediated gene transcription of both PAI-1 and Smad7 remained elevated greater than 2 fold (P>0.001; Figure 6A) in the presence of active Erk, three hours after removal of TGF-β treatment.

In addition to the described phosphorylation of nuclear translocated smads, Erk functions by phosphorylating a variety of cytoplasmic and nuclear targets, many of which are critical in cell cycle progression [39]–[42]. Having established a functional connection between Erk and the Smad signaling pathway, we sought to determine if Erk also plays a role in the proliferative effects of TGF-β in fibroblasts. The biological consequences of TGF-β induced Erk activation were addressed using a thymidine incorporation assay (Fig. 6B). Treatment with TGF-β yielded a 6 fold increase in DNA synthesis as compared to untreated fibroblasts. When cells were treated with U0126 to inhibit Erk activation prior to TGF-β addition, this growth stimulation was attenuated (P<0.001). Consistent with our previous data showing Pak2's role in Erk activation, expression of dnPak2 dramatically decreased TGF-β induced growth stimulation (Ad-EGFP+TGF-β vs Ad-dnPAK2+TGF-β; P<0.001). This reduction, rather than a complete block, may be due to the high endogenous Pak2 levels in these cells and dnPak2 being a competitive inhibitor over 20 h, in addition to the previously described minor contribution of Ras. Thus, the ability of TGF-β to induce replication in fibroblasts appears to depend primarily on the function of Pak2 acting through Erk.

## Discussion

TGF-β has previously been shown to mediate the activation of a number of downstream targets associated with PI3K signaling, in particular MAPKs such as Jnk and Erk [43]–[46]. However, a major limitation in our understanding of TGF-β biology is the lack of knowledge regarding a direct link between TGF-β “smad signaling” and the mechanisms and actions of the “non-smad” pathways in nontransformed (normal) cells. Considering the functional diversity that TGF-βs display, these pathway(s) have the potential to play a major role either as direct alternative signaling pathways or in crosstalk with smads, to generate the multitude of observed TGF-β effects. In this study we not only show that TGF-β stimulates both the Erk and smad pathways, but that the two pathways interact and are not independent (smad vs non-smad) as they modulate mesenchymal cellular responses to TGF-β.

Although different cell types exhibit different growth responses to TGF-β, much of the literature contains reports in which different cell types have been used within the same study to define TGF-β induced “non-smad” signaling. In our study we compared four phenotypically normal cell lines (2 fibroblast and 2 epithelial) treated with TGF-β. TGF-β was only able to induce Erk phosphorylation in fibroblasts, and not epithelial cells. It is important to note that this activation occurs with endogenous protein levels of the signal transduction pathway, in non-cancerous, non-transformed cells, using modest doses of TGF-β (2 ng/ml), demonstrating this is a normal response and not related to over-expression artifacts or cancer induced mutations. The same response was found in MEFs, indicating Erk activation is not limited to established cell lines, thus providing baseline data to compare TGF-β responses in other cell backgrounds. Also, in our studies fibroblasts were serum depleted, while epithelial cells were not, since serum depletion induces senescence. This was done in order to faithfully reflect the regulatory effects of TGF-β on each individual cell type. As a known effector of cell replication, it is interesting that Erk is activated in a cell type known to proliferate in response to TGF-β, but not a growth inhibited cell type, when using the appropriate cellular environment where these responses would occur.

To initiate TGF-β activation of the Erk signaling pathway, PI3K activation has been identified as necessary [12], [13]. Here we show that PI3K also acts downstream of the TGF-β receptor complex to induce the activation of Erk, through Pak2 phosphorylation of c-Raf at S338, a known site of Pak activation [34], [35], [47]. Unlike the canonical Ras/Raf/Erk pathway and data previously describing Erk activation by TGF-β [13], our study showed c-Raf activation appears to occur primarily through Pak2, similar to that described for Epo and PDGF [20], [21], and mimicing its budding yeast homologue, Ste20, by acting as a MAP4K [15]. Since TGF-β treatment of either Pak2-knockout or dnPak2 transfected cells caused a dramatic reduction, but not a complete block, of c-Raf or Erk phosphorylation or thymidine incorporation, this suggests that Ras may still be involved, but play a minor role. Beeser et al. [21] showed that neither EGF nor PDGF used only one pathway to activate Erk, but had a preference for either Ras or Pak, respectively. Our data concerrs with this, in that TGF-β appears to have a preference for Pak2 over Ras, but the two pathways work together to induce growth in fibroblasts through their coordinated activation of Erk.

The linker region of smads is believed to be important in regulating smad function [48], [49]. Previous studies have addressed the cross-talk between MAPKs and smad signaling [7], [18], [23], however an unambiguous definition remains elusive, primarily due to the lack of consistency in the cellular models used to define the interaction between the pathways. Using both subcellular fractionation and immunocytochemistry we demonstrated TGF-β induced, Erk mediated linker phosphorylated smad2 is found in the nucleus of fibroblasts. Although our data does not preclude the possibility of cytoplasmic Erk phosphorylation of smad linker region, followed by a rapid nuclear translocation, this seems unlike in that previously cytoplasmic smad2 linker phosphorylation has been shown to be associated with exclusion from the nucleus [18], [27]. Additionally, our data shows that in fibroblasts treated with TGF-β, Erk is the primary kinase responsible for smad-linker phosphorylation rather than CDK8 and 9 as shown in HaCat epithelial cells [25], since U0126 was able to completely abolish detectable smad2-linker phosphorylation. Kretzschmar et al. [18], showed oncogenic Ras and EGF, through Erk, resulted in nuclear exclusion of smad2/3, while others have shown [7], [23] Erk activity increased smad mediated transcription. Our results not only show Erk mediated linker region phosphorylation increased the half-life of receptor phosphorylated smad 2 and 3, but also an increase in duration of smad transcriptional activity. The difference between our data and Kretzchmar et al. [18] could be that our study defined fibroblast TGF-β signaling under endogenous conditions, while Kretzchmar et al. used epithelial cells and defined the interactions of overexpressed Ras and EGF pre-treatment with TGF-β signaling. Smads are rapidly phosphorylated through receptor serine/threonine kinase activity (within 30 min as shown in Figure 4B) and translocate into the nucleus, while TGF-β mediated Erk activation was much slower, resulting in smads being in the nucleus prior to significant Erk activation (C-terminal phospho-smads peak 30–60 min, while Erk is significantly above background 60–90 min. and increases through 4 h). By activating Erk, resulting in smad linker phosphorylation, either rapidly through EGF (as shown in Figure 4D) or constitutively through oncogenic Ras, Kretzchmar et al. may have changed the subcellular location where the interaction between smads 2/3 and Erk occurs, potentially altering the function of linker region phosphorylation to inhibiting smad2/3 from entering the nucleus as suggested by the Agonist/Antagonist interaction concept for Smad1 linker phosphorylation [25], [50]. Additionally, this demonstrates the complex effects of the growth factor mileu on a cell, in that a TGF-β/smad signal may be modulated by the presence of other growth factors acting on the PI3K pathway.

In summary, as depicted in figure 6C, we propose the following model; TGF-β induced phosphorylation of Erk begins with TGFβR activation of the PI3K/Pak2 pathway. As described in earlier studies [10], [51], [52], PI3K and smad activation differs in their subcellular location. Smads require clathrin mediated endocytosis prior to activation, while PI3K is activated from the plasma membrane. Although our data does not eliminate the role of Ras, it does indicate a subordinate role and the primary mechanism of c-Raf activation is through Pak2. Pak2 then activates c-Raf, followed by MEK and ultimately Erk. Nuclear translocated, activated Erk is able to phosphorylate the linker region of smad2/3 and increase their duration of transcriptional activity. In addition, activated Erk is able to stimulate cellular replication through its modulation of smad and other transcription factor's activity. The data presented here, defines the signaling pathway by which TGF-β results in the activation of Erk, in normal mesenchymal and not normal epithelial cells, thus providing a baseline to examine these differences in transformed cells. In addition, it clarifies and defines a role of Erk activation in modulating smad signaling and the consequences of the cross-talk between the two signaling pathways, suggesting that the concept of smad-dependent and smad-independent signaling pathways may be inappropriate. The findings that TGF-β/PI3K signaling in fibroblasts can play a major role in direct regulation of smad signaling indicates that TGF-β activates a complex, interacting signaling web rather than discrete smad/non-smad pathways.
